# ﻿Three new species of *Cyanosporus* (Polyporales, Basidiomycota) from China

**DOI:** 10.3897/mycokeys.107.126139

**Published:** 2024-08-09

**Authors:** Chao-Ge Wang, Shun Liu, Masoomeh Ghobad-Nejhad, Hong-Gao Liu, Yu-Cheng Dai, Yuan Yuan

**Affiliations:** 1 State Key Laboratory of Efficient Production of Forest Resources, School of Ecology and Nature Conservation, Beijing Forestry University, Beijing 100083, China Beijing Forestry University Beijing China; 2 Institute of Ecology and Key Laboratory for Earth Surface Processes of the Ministry of Education, College of Urban and Environmental Sciences, Peking University, Beijing 100871, China Peking University Beijing China; 3 Department of Biotechnology, Iranian Research Organization for Science and Technology (IROST), Tehran 3353-5111, Iran Iranian Research Organization for Science and Technology Tehran Iran; 4 Yunnan Key Laboratory of Gastrodia and Fungi Symbiotic Biology, Zhaotong University, Zhaotong 657000, China Zhaotong University Zhaotong China

**Keywords:** Brown rot, phylogeny, polypore, *Postia*, taxonomy, wood-decaying fungi

## Abstract

*Cyanosporus* is a cosmopolitan genus characterized by effused-reflexed to pileate basidiomata with a bluish tint and allantoid to cylindrical basidiospores which are negative to weakly positive in Melzer’s reagent and Cotton Blue, causing a brown rot. Three new species of *Cyanosporus*, namely, *C.linzhiensis*, *C.miscanthi* and *C.tabuliformis* are described and illustrated. Phylogenies on *Cyanosporus* are reconstructed with seven loci DNA sequences including ITS, nLSU, nSSU, mtSSU, RPB1, RPB2 and TEF1 based on phylogenetic analyses combined with morphological examination. The description for the new species is given. The main morphological characteristics of all 38 accepted species in *Cyanosporus* are summarized.

## ﻿Introduction

The genus *Cyanosporus* McGinty (Polyporales, Basidiomycota), typified by *C.caesius* (Schrad.) McGinty, was established by Lloyd (1909). It is characterized by annual, resupinate, effused-reflexed to pileate basidiomata; white, cream, bluish gray to pinkish buff pileal surface; white, cream, bluish gray to ash gray pore surface, hyphal system monomitic with generative hyphae clamped, and allantoid to cylindrical basidiospores, thin- to slightly thick-walled, with negative to weakly positive reaction in Melzer’s reagent and Cotton Blue, causing a brown rot in decayed wood (Papp 2014; Shen et al. 2019; Liu et al. 2021, 2022).

The type species of *Cyanosporus* (*P.caesius*, basionym: *Boletuscaesius*) was previously treated as *Polyporuscaesius* (Schrad.) Fr. (Fries 1821) and *Tyromycescaesius* (Schrad.) Murrill (Murrill 1907), the latter name was accepted by some mycologists (Donk 1960; Jahn 1963; Lowe 1975). Subsequently, the species was also transferred into *Spongiporus* Murrill, *Postia* Fr., *Oligoporus* Bref., respectively (David 1980; Jülich 1982; Gilbertson and Ryvarden 1985), because *Postia* has priority over *Spongiporus* and *Oligoporus*, and *Postiacaesia* was widely accepted (Papp 2014).

*Cyanosporus* species were included in *Postia* Fr. typified by *Postialactea* (Fr.) P. Karst. Molecular analysis (Ţura et al. 2008; Pildain and Rajchenberg 2012; Ortiz-Santana et al. 2013) clustered the *Postia* species in two clades, one of which included *Postiacaesia* (Schrad.) P. Karst. Several studies acknowledged the morphological variability of *P.caesia*, and its closely related taxa referred to as *P.caesia* complex (Ţura et al. 2008; Pildain and Rajchenberg 2012; Papp 2014). Papp (2014) discussed the taxonomic status of the *Postiacaesia* complex, and proposed a subgenus, Postiasubg.Cyanosporus (McGinty) V. Papp for including the complex (involved five species: *P.alni*, *P.caesia*, *P.luteocaesia*, *P.mediterraneocaesia*, *P.subcaesia*). Miettinen et al. (2018) studied the *Postiacaesia* complex based on phylogenetic and morphological analyses, selected a neotype of *P.caesia* (LY BR-6776 collected from Germany) from type locality and described ten new species in *P.caesia* complex. A recent molecular study on *Postia* s.l. and related genera in Shen et al. (2019), considered *Cyanosporus* and *Postia* were two different genera. Two separate species were addressed in the family Postiaceae (Liu et al. 2023). Morphologically both genera differ by the more or less bluish basidiocarps and weakly cyanophilous basidiospores in *Cyanosporus*, while in *Postia*, basidiocarps lack a blue tint and basidiospores are acyanophilous (Shen et al. 2019; Liu et al. 2021, 2022, 2023).

Up to now, 35 species have been accepted in *Cyanosporus*, 23 of which are distributed in China (Liu et al. 2022). During the studies on Chinese polyporoid fungi causing brown rot, some samples were collected from southwest and northern China that morphologically correspond to *Cyanosporus*. The objective of this study is to confirm the identity of these specimens, through a phylogenetic analysis based on a seven loci dataset (ITS+nLSU+mtSSU+nuSSU+RPB1+RPB2+TEF1), and to describe and illustrate the new species.

## ﻿Materials and methods

### ﻿Morphological studies

The studied specimens are deposited in the Fungarium of the Institute of Microbiology, Beijing Forestry University (BJFC). Morphological descriptions are based on field notes and voucher specimens. The microscopic analysis follows Miettinen et al. (2018) and Wu et al. (2022a). Sections were studied at a magnification of up to 1000× using a Nikon Eclipse 80i microscope and phase contrast illumination. Description of microscopic features and measurements was made from slide preparations stained with KOH, Cotton Blue and Melzer’s reagent. Basidiospores were measured from sections cut from the tubes. To represent the variation in the size of spores, 5% of measurements were excluded from each end of the range and are given in parentheses. In the description: KOH = 5% potassium hydroxide, IKI = Melzer’s reagent, IKI– = neither amyloid nor dextrinoid, CB = Cotton Blue, CB– = acyanophilous in Cotton Blue, L = arithmetic average of spore length, W = arithmetic average of spore width, Q = L/W ratios, and n = number of basidiospores/measured from given number of specimens. Color terms follow Anonymous (1969) and Petersen (1996).

### ﻿DNA extraction, amplification and sequencing

A CTAB rapid plant genome extraction kit-DN14 (Aidlab Biotechnologies Co., Ltd, Beijing) was used to obtain DNA from dried specimens, followed by the polymerase chain reaction (PCR) according to the manufacturer’s instructions with some modifications (Shen et al. 2019; Sun et al. 2020). The internal transcribed spacer (ITS) and large subunit nuclear ribosomal RNA gene (nLSU) were amplified using the primer pairs ITS5/ITS4 and LR0R/LR7 (White et al. 1990; Hopple and Vilgalys 1999) (https://sites.duke.edu/vilgalyslab/rdna_primers_for_fungi/). The small subunit mitochondrial ribosomal DNA (mtSSU) region was amplified with primer pairs MS1 and MS2 (White et al. 1990). The small subunit nuclear ribosomal RNA gene (nSSU) region was amplified with primer pairs NS1 and NS4 (White et al. 1990). Part of TEF1 was amplified with primer pairs EF1-983F and EF1-1567R (Rehner and Buckley 2005). The RPB1 was amplified with primer pairs RPB1-Af and RPB1-Cr (Matheny et al. 2002). The RPB2 was amplified with primer pairs fRPB2-5F and fRPB2-7CR (Matheny 2005).

The PCR procedure for ITS, mtSSU and TEF1 was as follows: initial denaturation at 95 °C for 3 min, followed by 34 cycles at 94 °C for 40 s, 54 °C for ITS, 58 °C for mtSSU, and 54 °C for TEF for 45 s and 72 °C for 1 min, and a final extension of 72 °C for 10 min. The PCR procedure for nLSU and nSSU was as follows: initial denaturation at 94 °C for 1 min, followed by 34 cycles of denaturation at 94 °C for 30 s, annealing at 50 °C for nLSU and 52 °C for nSSU for 1 min and extension at 72 °C for 1.5 min, and a final extension at 72 °C for 10 min. The PCR procedure for RPB1 and RPB2 was initial denaturation at 94 °C for 2 min, followed by 10 cycles at 94 °C for 45 s, 60 °C for 45 s and 72 °C for 1.5 min, then followed by 37 cycles at 94 °C for 45 s, 52 °C for 1 min and 72 °C for 1.5 min, and a final extension of 72 °C. The PCR products were purified and sequenced at the Beijing Genomics Institute (BGI), China, with the same primers as used in PCR. Newly generated sequences were deposited in GenBank. All sequences analysed in this study are listed in Table 1.

**Table 1.** Species, specimens, and GenBank accession number of sequences used for phylogenetic analyses in this study.

Species	Specimen voucher	Country	GenBank accession NO.						
			ITS	LSU	mtSSU	nrSSU	RPB1	RPB2	TEF1
*Amaropostiahainanensis* B.K. Cui, L.L. Shen & Y.C. Dai	Cui 13739 (holotype)	China	KX900909	KX900979	KX901053	KX901123	KX901171	KX901223	–
*A.stiptica* (Pers.) B.K. Cui, L.L. Shen & Y.C. Dai	Cui 10043	China	KX900906	KX900976	KX901046	KX901119	KX901167	KX901219	–
*Amylocystislapponica* (Romell) Bondartsev & Singer	HHB-13400	USA	KC585237	KC585059	–	–	–	–	–
*A.lapponica*	OKM-4418	USA	KC585238	KC585060	–	–	–	–	–
*Antrodiaserpens* (Fr.) P. Karst.	Dai 7465	Luxemburg	KR605813	KR605752	KR606013	KR605913	–	KR610832	KR610742
*A.tanakae* (Murrill) Spirin & Miettinen	Cui 9743	China	KR605814	KR605753	KR606014	KR605914	–	KR610833	KR610743
*Calcipostiaguttulata* (Sacc.) B.K. Cui, L.L. Shen & Y.C. Dai	Cui 10018	China	KF727432	KJ684978	KX901065	KX901138	KX901181	KX901236	KX901276
*C.guttulata*	Cui 10028	China	KF727433	KJ684979	KX901066	KX901139	KX901182	KX901237	KX901277
*Cyanosporusalni* (Niemelä & Vampola) B.K. Cui, L.L. Shen & Y.C. Dai	H 7019137 (holotype)	Slovakia	MG137026	–	–	–	–	–	–
*C.alni*	Cui 7185	China	KX900879	KX900949	KX901017	KX901092	KX901155	KX901202	KX901254
*C.alni*	Dai 14845	Poland	KX900880	KX900950	KX901018	KX901093	KX901156	KX901203	KX901255
*C.arbuti* (Spirin) B.K. Cui & Shun Liu	Spirin 8327 (holotype)	USA	MG137039	–	–	–	–	–	MG137132
*C.auricomus* (Spirin & Niemelä) B.K. Cui & Shun Liu	Cui 13518	China	KX900887	KX900957	KX901025	KX901100	–	KX901209	–
*C.auricomus*	Cui 13519	China	KX900888	KX900958	KX901026	KX901101	–	–	–
*C.auricomus*	TN 8310 (holotype)	Finland	MG137040	–	–	–	–	–	–
*C.bifarius* (Spirin) B.K. Cui & Shun Liu	Spirin 6402 (holotype)	Russia	MG137043	–	–	–	–	–	MG137133
*C.bifarius*	Cui 17534	China	OL423598	OL423608	OL437195	OL423620	OL444985	OL446999	OL444994
*C.bifarius*	Cui 16277	China	OL423599	OL423609	OL437196	OL423621	OL444986	OL447000	OL444995
*C.bubalinus* B.K. Cui & Shun Liu	Cui 16976	China	MW182172	MW182225	MW182208	MW182189	MW191547	MW191563	MW191530
*C.bubalinus*	Cui 16985 (holotype)	China	MW182173	MW182226	MW182209	MW182190	MW191548	MW191564	MW191531
*C.caesiosimulans* (G.F. Atk.) B.K. Cui & Shun Liu	Spirin 4199	Russia	MG137061	–	–	–	–	–	MG137140
*C.caesiosimulans*	Miettinen 16976 (holotype)	USA	MG137054	–	–	–	–	–	MG137137
*C.caesius* (Schrad.) McGinty	Schuster 51 (neotype)	Germany	MG137045	–	–	–	–	–	–
*C.caesius*	Miettinen 14156	Finland	MG137048	–	–	–	–	–	MG137134
*C.caesius*	Cui 18630	France	OL423600	OL423610	OL437197	OL423622	–	–	OL444996
C.aff.caesius	K 32713	UK	AY599576	–	–	–	–	–	–
C.aff.caesius	K 32425	UK	AY599575	–	–	–	–	–	–
*C.coeruleivirens* (Corner) B.K. Cui, Shun Liu & Y.C. Dai	Miettinen 12214	Indonesia	MG137063	–	–	–	–	–	–
*C.coeruleivirens*	Dai 19220	China	MW182174	MW182227	MW182210	MW182191	MW191549		MW191532
*C.comatus* (Miettinen) B.K. Cui & Shun Liu	Miettinen 14755,1 (holotype)	USA	MG137066	–	–	–	–	–	–
*C.cyanescens* (Miettinen) B.K. Cui & Shun Liu	Miettinen 13602 (holotype)	Finland	MG137067	–	–	–	–	–	MG137142
*C.cyanescens*	Miettinen 15919.2	Spain	MG137071	–	–	–	–	–	MG137144
*C.flavus* B.K. Cui & Shun Liu	Cui 18547	China	MW448564	MW448561	–	MW448557	MW452596	MW452599	MW452601
*C.flavus*	Cui 18562 (holotype)	China	MW448565	MW448562	–	MW448558	MW452597	MW452600	MW452602
*C.fusiformis* B.K. Cui, L.L. Shen & Y.C. Dai	Cui 10775	China	KX900868	KX900938	KX901006	KX901081	–	KX901191	KX901245
*C.fusiformis*	Dai 15036 (holotype)	China	KX900867	KX900937	KX901005	KX901080	–	KX901190	KX901244
*C.glaucus* (Spirin & Miettinen) B.K. Cui & Shun Liu	Spirin 5317	Russia	MG137078	–	–	–	–	–	–
*C.glaucus*	Spirin 6580 (holotype)	Russia	MG137081	–	–	–	–	–	MG137145
*C.gossypinus* (Moug. & Lév.) B.K. Cui & Shun Liu	Rivoire 6658 (topotype)	France	–	–	–	–	–	–	MG137146
*C.hirsutus* B.K. Cui & Shun Liu	Cui 17083 (holotype)	China	MW182179	MW182233	MW182214	MW182197	MW191554	MW191568	MW191538
*C.hirsutus*	Cui 17343	China	OL423601	OL423611	OL437198	OL423623	OL444987	OL447001	OL444997
*C.hirsutus*	Cui 17342	China	OL423602	OL423612	OL437199	OL423624	OL444988	OL447002	OL444998
***C.linzhiensis***	**Dai 27141**	**China**	**PP479781 ^a^**	**PP479803** ^a^	**PP510196 ^a^**	**PP488288 ^a^**	**PP526258 ^a^**	**PP526267 ^a^**	–
***C.linzhiensis***	**Dai 27023 (holotype)**	**China**	**PP479782 ^a^**	**PP479804** ^a^	**PP510197** ^a^	**PP488289 ^a^**	**PP526259 ^a^**	**PP526268 ^a^**	–
*C.livens* (Miettinen & Vlasák) B.K. Cui & Shun Liu	Spirin 8728	USA	MG137090	–	–	–	–	–	MG137150
*C.livens*	Miettinen 17177 (holotype)	USA	MG137082	–	–	–	–	–	MG137147
*C.luteocaesius* (A. David) B.K. Cui, L.L. Shen & Y.C. Dai	Rivoire 2605 (topotype)	France	MG137091	–	–	–	–	–	–
*C.magnus* (Miettinen) B.K. Cui & Shun Liu	Dai 21105	China	OL423603	OL423613	OL437200	OL423625	OL444989	OL447003	OL444999
*C.magnus*	Cui 16983	China	MW182180	MW182234	MW182215	MW182198	MW191555	MW191569	MW191539
*C.magnus*	Miettinen 10634 (holotype)	China	KC595944	KC595944	–	–	–	–	MG137151
*C.mediterraneocaesius* (M. Pieri & B. Rivoire) B.K. Cui, L.L. Shen & Y.C. Dai	LY BR 4274	France	KX900886	–	KX901024	KX901099	–	–	–
*C.microporus* B.K. Cui, L.L. Shen & Y.C. Dai	Cui 11014 (holotype)	China	KX900878	KX900948	KX901016	KX901091	–	KX901201	–
*C.microporus*	Dai 11717	China	KX900877	KX900947	KX901015	KX901090	–	KX901200	–
***C.miscanthi***	**Dai 26684**	**China**	**PP479784 ^a^**	**PP479806 ^a^**	**PP510199 ^a^**	**PP488291 ^a^**	**PP526261 ^a^**	**PP526270 ^a^**	**PP526276 ^a^**
***C.miscanthi***	**Dai 26687 (holotype)**	**China**	**PP479786 ^a^**	**PP479808 ^a^**	**PP510201 ^a^**	**PP488293 ^a^**	**PP526263 ^a^**	**PP526272 ^a^**	**PP526277 ^a^**
***C.miscanthi***	**Dai 26689**	**China**	**PP479783 ^a^**	**PP479805 ^a^**	**PP510198 ^a^**	**PP488290 ^a^**	**PP526260 ^a^**	**PP526269 ^a^**	**PP526275 ^a^**
***C.miscanthi***	**Dai 26695**	**China**	**PP479787 ^a^**	**PP479809 ^a^**	**PP510202 ^a^**	**PP488294 ^a^**	**PP526264 ^a^**	**PP526273 ^a^**	**PP526278 ^a^**
***C.miscanthi***	**Dai 26701**	**China**	**PP479785 ^a^**	**PP479807 ^a^**	**PP510200 ^a^**	**PP488292 ^a^**	**PP526262 ^a^**	**PP526271 ^a^**	–
*C.nothofagicola* B.K. Cui, Shun Liu & Y.C. Dai	Cui 16697 (holotype)	Australia	MW182181	MW182235	MW182216	MW182199	MW191556	MW191570	MW191540
*C.nothofagicola*	Dai 18765	Australia	MW182182	MW182236	MW182217	MW182200	MW191557	–	MW191541
*C.piceicola* B.K. Cui, L.L. Shen & Y.C. Dai	Cui 10626 (holotype)	China	KX900862	KX900932	KX901001	KX901075	–	KX901185	–
*C.piceicola*	Cui 12158	China	KX900866	KX900936	KX901004	KX901079	KX901153	KX901189	KX901243
*C.populi* (Miettinen) B.K. Cui & Shun Liu	Miettinen 17043 (holotype)	USA	MG137092	–	–	–	–	–	MG137153
*C.populi*	Cui 17087a	China	MW182183	MW182237	MW182218	MW182201	MW191558	MW191571	MW191542
*C.populi*	Dai 18934	China	OL423604	OL423614	OL437201	OL423626	OL444990	OL447004	OL445000
*C.populi*	Cui 17557	China	OL423605	OL423615	OL437202	OL423627	OL444991	OL447005	OL445001
*C.rigidus* B.K. Cui & Shun Liu	Cui 17032 (holotype)	China	OL423606	OL423617	OL437204	OL423629	OL444993	–	OL445003
*C.simulans* (P. Karst.) B.K. Cui & Shun Liu	Miettinen 20422	Finland	MG137110	–	–	–	–	–	MG137160
*C.simulans*	TN 8846 (holotype)	Finland	MG137103	–	–	–	–	–	–
*C.subcaesius* (A. David) B.K. Cui, L.L. Shen & Y.C. Dai	JV 0110/24	Czechia	MG137117	–	–	–	–	–	MG137164
*C.subcaesius*	Alix David 652 (isotype)	France	MG137116	–	–	–	–	–	–
*C.subhirsutus* B.K. Cui, L.L. Shen & Y.C. Dai	Cui 11330	China	KX900873	KX900943	KX901011	KX901086	–	KX901196	KX901250
*C.subhirsutus*	Dai 14892 (holotype)	China	KX900871	KX900941	KX901009	KX901084	–	KX901194	KX901248
*C.submicroporus* B.K. Cui & Shun Liu	Cui 16306	China	MW182184	MW182239	MW182220	MW182203	MW191560	MW191573	MW191544
*C.submicroporus*	Cui 18156 (holotype)	China	MW182186	MW182241	MW182222	MW182205	–	MW191574	–
*C.subungulatus* B.K. Cui & Shun Liu	Cui 18046 (holotype)	China	MW448566	MW448563	MW448560	MW448559	MW452598	–	MW452603
*C.subungulatus*	Zhao 10833	China	MW742586	OL423616	OL437203	OL423628	OL444992	–	OL445002
*C.subviridis* (Ryvarden & Guzmán) B.K. Cui & Shun Liu	Spirin 8774a	USA	MG137120	–	–	–	–	–	MG137166
*C.subviridis*	Penttilä 14376	Finland	–	–	–	–	–	–	MG137165
***C.tabuliformis***	**Dai 26063 (holotype)**	**China**	**PP479788 ^a^**	**PP479810 ^a^**	**PP510203 ^a^**	**PP488295 ^a^**	**PP526265 ^a^**	**PP526274 ^a^**	**PP526279 ^a^**
***C.tabuliformis***	**Dai 26066**	**China**	**PP479789 ^a^**	**PP479811 ^a^**	**PP510204 ^a^**	**PP488296 ^a^**	**PP526266 ^a^**	–	**PP526280 ^a^**
*C.tenuicontextus* B.K. Cui & Shun Liu	Cui 16280 (holotype)	China	OL423607	OL423618	OL437205	OL423630	–	–	OL445004
*C.tenuicontextus*	Zhao 813	China	MG231802	OL423619	OL437206	OL423631	–	–	OL445005
*C.tenuis* B.K. Cui, Shun Liu & Y.C. Dai	Cui 10788 (holotype)	China	KX900885	KX900955	KX901023	KX901098	KX901161	KX901208	–
*C.tenuis*	Dai 12974	China	KX900884	KX900954	KX901022	KX901097	KX901160	KX901207	KX901258
*C.tricolor* B.K. Cui, L.L. Shen & Y.C. Dai	Cui 12233 (holotype)	China	KX900876	KX900946	KX901014	KX901089	–	KX901199	KX901253
*C.tricolor*	Cui 10790	China	KX900875	KX900945	KX901013	KX901088	–	KX901198	KX901252
*C.ungulatus* B.K. Cui, L.L. Shen & Y.C. Dai	Cui 10778	China	KX900870	KX900940	KX901008	KX901083	–	KX901193	KX901247
*C.ungulatus*	Dai 12897 (holotype)	China	KX900869	KX900939	KX901007	KX901082	KX901154	KX901192	KX901246
*C.yanae* (Miettinen & Kotir.) B.K. Cui & Shun Liu	Kotiranta 27606	Russia	MG137122	–	–	–	–	–	MG137168
*C.yanae*	Kotiranta 27454 (holotype)	Russia	MG137121	–	–	–	–	–	MG137167
*Cystidiopostiahibernica* (Berk. & Broome) B.K. Cui, L.L. Shen & Y.C. Dai	Cui 2658	China	KX900905	KX900975	KX901045	KX901118	–	KX901218	–
*C.inocybe* (A. David & Malençon) B.K. Cui, L.L. Shen & Y.C. Dai	LY BR 3703	France	KX900903	KX900973	KX901044	KX901116	–	–	KX901267
*C.pileata* (Parmasto) B.K. Cui, L.L. Shen & Y.C. Dai	Cui 10034	China	KX900908	KX900956	KX901050	KX901122	KX901170	KX901222	KX901269
*Fuscopostiaduplicate* (L.L. Shen, B.K. Cui & Y.C. Dai) B.K. Cui, L.L. Shen & Y.C. Dai	Dai 13411 (holotype)	China	KF699125	KJ684976	KR606027	KR605928	KX901174	KR610845	KR610756
*F.fragilis* (Fr.) B.K. Cui, L.L. Shen & Y.C. Dai	JV 0610/8	Czechia	JF950573	–	–	–	–	–	–
*F.leucomallella* (Murrill) B.K. Cui, L.L. Shen & Y.C. Dai	Cui 9599	China	KF699123	KJ684983	KX901056	KX901129	KX901176	KX901228	KX901272
*Jahnoporusbrachiatus* Spirin, Vlasák & Miettinen	X 3232	Russia	KU165781	–	–	–	–	–	–
*J.hirtus* (Cooke) Nuss	Spinosa 10 X 2014	USA	KU165784	–	–	–	KY949044	–	–
*J.oreinus* Spirin, Vlasák & Miettinen	X 3241	Russia	KU165785	–	–	–	–	–	–
*Oligoporusrennyi* (Berk. & Broome) Donk	TN-6645	Finland	KC595929	KC595929	–	–	–	–	–
*O.sericeomollis* (Romell) Bondartseva	Cui 9870	China	KX900920	KX900990	KX901068	KX901141	KX901184	–	–
*Osteinaobducta* (Berk.) Donk	Cui 10074	China	KX900924	KX900994	KX901071	KX901144	–	KX901240	–
*O.undosa* (Peck) Zmitr.	Dai 7105	China	KX900921	KX900991	KX901069	KX901142	–	KX901238	–
*Postiaamurensis* Y.C. Dai & Penttilä	Dai 903 (holotype)	China	KX900901	KX900971	KX901042	–	–	–	–
*P.hirsuta* L.L. Shen & B.K. Cui	Cui 11237 (holotype)	China	KJ684970	KJ684984	KX901038	KX901113	–	–	KX901266
*P.lactea* (Fr.) P. Karst.	Cui 12141	China	KX900892	KX900962	KX901029	KX901104	KX901163	KX901211	KX901260
*P.lowei* (Pilát) Jülich	Cui 9585	China	KX900898	KX900968	KX901035	KX901110	–	–	–
*P.ochraceoalba* L.L. Shen, B.K. Cui & Y.C. Dai	Cui 10802 (holotype)	China	KM107903	KM107908	KX901041	KX901115	–	KX901216	–
*P.sublowei* B.K. Cui, L.L. Shen & Y.C. Dai	Cui 9597 (holotype)	China	KX900900	KX900970	KX901037	KX901112	–	–	KX901265
*P.tephroleuca* (Fr.) Jülich	Dai 12610	Finland	KX900897	KX900967	KX901034	KX901109	KX901166	KX901214	KX901263
*Resupinopostialateritia* (Renvall) B.K. Cui, L.L. Shen & Y.C. Dai	Dai 2652	China	KX900913	KX900983	–	–	–	–	–
*R.sublateritia* B.K. Cui & Shun Liu	Dai 22760	China	OQ476281	OQ476340	OQ476447	OQ476396	OQ506088	OQ511187	OQ511241
*Spongiporusbalsameus* (Peck) A. David	Cui 9835	China	KX900916	KX900986	KX901061	KX901134	–	KX901233	–
*S.leucospongia* (Cooke & Harkn.) Murrill	JV 0709/123	USA	–	KX900988	KX901064	KX901137	–	–	KX901275
*S.floriformis* (Quél.) Zmitr.	Cui 10292	China	KM107899	KM107904	KX901058	KX901131	KX901178	KX901230	KX901274

^a^ Newly generated sequences in this study. Bold = new taxa and sequences generated in this study.

### ﻿Sequence alignment

Sequences generated from this study were aligned with additional sequences downloaded from GenBank using BioEdit (Hall 1999) and ClustalX (Thompson et al. 1997). The final ITS, nLSU, mtSSU, nSSU, RPB1, RPB2 and TEF1 datasets were subsequently aligned using MAFFT v.7 under the E-INS-i strategy with no cost for opening gaps and equal cost for transformations (command line: mafft –genafpair –maxiterate 1000) (Katoh and Standley 2013) and visualized in BioEdit. Alignments were spliced and transformed formats in Mesquite v.3.2. (Maddison and Maddison 2017). Multiple sequence alignments were trimmed by trimAI v.1.2 using the -htmlout-gt 0.8 -st option to deal with gaps when necessary (Capella-Gutierrez et al. 2009).

### ﻿Phylogenetic analyses

In this study, a seven loci dataset (ITS+LSU+mtSSU+nrSSU+RPB1+RPB2+TEF1) was used to reconstruct the phylogenetic position of the new species. The sequence alignment and the retrieved topology were deposited in TreeBase (http://www.treebase.org), under accession ID: 31280 (Reviewer access URL: http://purl.org/phylo/treebase/phylows/study/TB2:S31280?x-access-code=605c3765137c8814e37dd70c560cb4de&format=html). Sequences of *Antrodiaserpens* (Fr.) P. Karst. and *Antrodiatanakae* (Murrill) Spirin & Miettinen, obtained from GenBank, were used as the outgroups (Liu et al. 2021). The phylogenetic analyses followed the approach of Han et al. (2016) and Zhu et al. (2019). Maximum parsimony (MP), Maximum Likelihood (ML), and Bayesian Inference (BI) analyses were performed based on one dataset. The best-fit evolutionary model was selected by Akaike Information Criterion (AIC) in MrModeltest 2.2 (Nylander 2004) after scoring 24 models of evolution in PAUP* version 4.0b10 (Swofford 2002).

The MP topology and bootstrap values (MP-BS) obtained from 1000 replicates were computed in PAUP* version 4.0b10 (Swofford 2002). All characters were equally weighted, and gaps were treated as missing. Trees were inferred using the heuristic search option with TBR branch swapping and 1000 random sequence additions. Maxtrees were set to 5,000 branches of zero length were collapsed, and all parsimonious trees were saved. Descriptive tree statistics tree length (TL), composite consistency index (CI), retention index (RI), rescaled consistency index (RC), and homoplasy index (HI) were calculated for each maximum parsimonious tree (MPT) generated. Sequences were also analysed using Maximum Likelihood (ML) with RAxML-HPC2 through the CIPRES Science Gateway (Miller et al. 2010). Branch support (BT) for ML analysis was determined by 1 000 bootstrap replicates. Bayesian phylogenetic inference and Bayesian Posterior Probabilities (BPP) were computed with MrBayes 3.1.2 (Ronquist and Huelsenbeck 2003). Four Markov chains were run for 3.5 M generations until the split deviation frequency value was less than 0.01, and trees were sampled every 100 generations. The first 25% of the sampled trees were discarded as burn-in and the remaining ones were used to reconstruct a majority rule consensus and calculate Bayesian Posterior Probabilities (BPP) of the clades. All trees were viewed in FigTree v. 1.4.3 (http://tree.bio.ed.ac.uk/software/figtree/). Branches that received bootstrap support MP ≥ 75%, ML ≥ 75%, and BPP ≥ 0.95 were considered as significantly supported. The MP and ML bootstrap supports ≥ 50% and BBP ≥ 0.90 are presented on topologies from ML analysis, respectively.

## ﻿Results

### ﻿Molecular phylogeny

**Figure 1.**
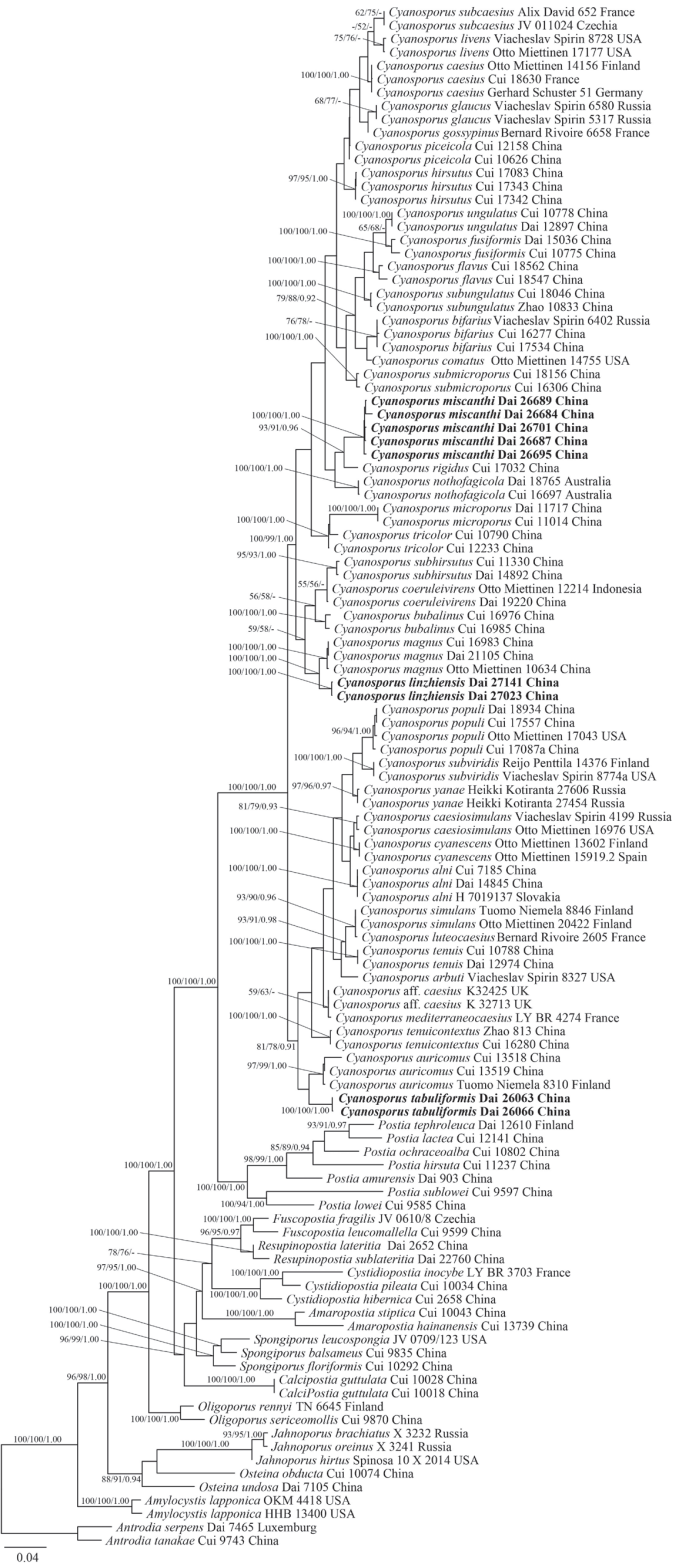
A Maximum Likelihood phylogenetic tree of *Cyanosporus* based on a dataset of ITS+nLSU+mtSSU+nuSSU+RPB1+RPB2+TEF1. ML bootstrap values higher than 50% and Bayesian posterior probabilities values more than 0.90 are shown. New taxa are in bold.

The combined seven loci dataset (ITS+LSU+mtSSU+nrSSU+RPB1+RPB2+TEF1) included sequences from 115 samples representing 68 species. The dataset had an aligned length of 5639 characters, of which 3800 (56%) characters are constant, 345 (9%) are variable and parsimony-uninformative and 1494 (35%) are parsimony informative. Maximum parsimony analysis yielded eleven equally-parsimonious tree (TL = 6767, CI = 0.414, RI = 0.701, RC = 0.290, HI = 0.586). The phylogenetic reconstruction performed with Maximum Likelihood (ML) and Bayesian Inference (BI) analyses showed similar topology and few differences in statistical support. The best model-fit applied in the Bayesian analysis was GTR+I+G, lset nst = 4, rates = invgamma, and prset statefreqpr = dirichlet (1, 1, 1, 1). Bayesian analysis resulted in a nearly congruent topology with an average standard deviation of split frequencies = 0.008647 to ML analysis, and thus only the ML tree is provided (Fig. 1). The phylogeny (Fig. 1) confirmed *Cyanosporus* and *Postia* as two independent and closely related clades with full support (100% MP, 100% ML, 1.00 BPP). Sequences of three new species, viz. *C.linzhiensis*, *C.miscanthi*, *C.tabuliformis*, were placed in three fully supported and independent lineages in *Cyanosporus* clade (Fig. 1). Though the *Cyanosporus* clade formed two subclades, the supports were at a very weak rate and species in the two subclades share similar characteristics. So the morphological characters cannot explain this separation.

### ﻿Taxonomy

The main morphological characteristics of the accepted species in *Cyanosporus* are provided in Table 2.

**Table 2.** The main morphological characteristics of the accepted species in *Cyanosporus*.

Species	Type locality	Basidiomata	Upper surface	Color of poroid surface	Amyloid (greenish in IKI) tramal hyphae	Shape of basidiospores	Size of Basidiospores (μm)	Cyanophilous basidiospores	References
*C.alni*	Slovakia	Annual, Pileate to rarely effused-reflexed	Cream, ochraceous to brownish with a bluish-grayish tint; velutinate	White to cream, in older and dry specimens with a light bluish-grayish tint	–	Allantoid to very narrow cylindrical	4.4–6 × 1.1–1.3	–	Niemelä et al. (2001); Miettinen et al. (2018)
*C.arbuti*	USA	Annual, Pileate to effused-reflexed	White to pale cream; glabrous	White to cream, in older and dry specimens with a light bluish-grayish tint	–	Allantoid	4.1–5.1 × 1–1.2	+	Miettinen et al. (2018)
*C.auricomus*	Finland	Annual, Pileate	White to cream, yellowish to bright yellow, in older specimens with pale to dark ochraceous; hirsute	Bright yellow, green when bruised, then with an ochraceous tint	+	Allantoid	4.4–5.6 × 1.5–1.8	+	Miettinen et al. (2018)
*C.bifarius*	Russia	Annual, Pileate	Light gray, then with an ochraceous tint; velutinate	White to cream, in older and dry specimens with a light ochraceous tint	–	Allantoid	3.7–4.4 × 1–1.2	+	Miettinen et al. (2018)
*C.bubalinus*	China	Annual, Pileate	White to cream when fresh, cream to pinkish buff when dry; tomentose	White to cream when fresh, straw yellow to buff when dry	–	Cylindrical, slightly curved	4.3–4.8 × 1.2–1.7	–	Liu et al. (2021)
*C.caesiosimulans*	USA	Annual, Pileate to effused-reflexed	White to cream, then grayish to pale ochraceous with very rarely with bluish flecks or faint zones; glabrous	White to cream, in older and dry specimens with a light bluish-grayish tint	–	Allantoid	4.2–5.5 × 1.1–1.4	+	Miettinen et al. (2018)
*C.caesius*	Germany	Annual, Pileate to effused-reflexed	Grayish to bluish when fresh, blue when bruised; hirsute	White to pale gray when fresh, bluish when bruised	+	Cylindrical to allantoid	4.5–6 × 1.5–2	+	Ryvarden and Gilbertson (1994)
*C.coeruleivirens*	Malaysia	Annual, Pileate	White to bluish green; velutinate	White when fresh, bluish green when bruised	–	Allantoid	4–5 × 1–1.3	+	Corner (1989)
*C.comatus*	USA	Annual, Pileate to effused-reflexed	Cream to pale ochraceous; velutinate	Cream, in older and dry specimens with a bluish-grayish tint	–	Allantoid	4.1–4.9 × 1.1–1.3	+	Miettinen et al. (2018)
*C.cyanescens*	Finland	Annual, Pileate to reraly effused-reflexed	White to pale ochraceous, then pale ochraceous, rarely with a bluish-grayish tint; glabrous	White to cream, in older and dry specimens with a light bluish-grayish tint	–	Allantoid	4.7–6.1 × 1.1–1.6	+	Miettinen et al. (2018)
*C.flavus*	China	Annual, Pileate	Ash-gray to light vinaceous gray when fresh, pale mouse-gray to mouse-gray when dry; hirsute	White to cream when fresh, buff to lemon-chrome when dry	–	Slim allantoid	4.6–5.2 × 0.8–1.3	–	Liu et al. (2022)
*C.fusiformis*	China	Annual, Pileate to effused-reflexed	White to cream, with a blue tint at the center when fresh, vinaceous gray to dark gray when dry; tomentose	White when fresh, buff to clay buff when dry	–	Slim allantoid	4.5–5.2 × 0.8–1.1	–	Shen et al. (2019)
*C.glaucus*	Russia	Annual, Pileate	Grayish, plumbeous to bluish gray or grayish-brown; hirsute	White to cream when fresh, in older and dry specimens with a light bluish-grayish tint	+	Allantoid	4.1–5.4 × 1.1–1.5	+	Miettinen et al. 2018
*C.gossypinus*	France	Annual, Pileate	Cream to light gray; glabrous	Cream to bluish-grayish	–	Cylindrical to allantoid	4.1–5.1 × 1.2–1.7	+	Miettinen et al. (2018)
*C.hirsutus*	China	Annual, Pileate	Ash-gray to light grayish brown with bluish gray zones when fresh, grayish to grayish brown when dry; hirsute	Cream when fresh, straw yellow to olivaceous buff when dry	–	Cylindrical, slightly curved	4–4.7 × 1.2–1.5	–	Liu et al. (2021)
***C.linzhiensis***	China	Annual, pileate	White with a blue tint when fresh, becoming white to pinkish buff when dry; velutinate	White to pale bluish gray when fresh, pinkish buff to honey yellow and with a blue tint when dry	–	Allantoid	4–5 × 1.2–1.5	–	This study
*C.livens*	USA	Annual, Pileate	Cream, plumbeous to bluish gray to ochraceous; velutinate	Cream, in older and dry specimens with a light bluish-grayish tint	–	Cylindrical to allantoid	4.1–5.7 × 1.1–1.5	+	Miettinen et al. (2018)
*C.luteocaesius*	France	Annual, resupinate to effused-reflexed	White to yellow when fresh, brownish when dry; tomentose	Yellow when fresh, with a light bluish tint when bruised	+	Allantoid	5–6 × 2	+	Ryvarden and Gilbertson (1994)
*C.magnus*	China	Annual, Pileate	White when fresh, cream to light grayish and ochraceous when dry; velutinate	White when fresh, ochraceous with a bluish tint when dry	–	Allantoid	3.6–4.4 × 1–1.2	+	Miettinen et al. (2018)
*C.mediterraneocaesius*	France	Annual, effused-reflexed to resupinate	White to cream or pale ochraceous; velutinate	White to cream, in older and dry specimens pale ochraceous, with a light bluish-grayish tint	–	Cylindrical to allantoid	4.2–5.8 × 1.3–1.7	+	Miettinen et al. (2018)
*C.microporus*	China	Annual, Pileate	White to cream with blue tint when fresh, cream to pinkish-buff when dry; velutinate	White when fresh, bluish when bruised, cream to buff when dry	–	Allantoid	4.5–4.9 × 1–1.2	–	Shen et al. (2019)
***C.miscanthi***	China	Annual, Pileate to effused-reflexed	White to pale bluish gray when fresh and dry; velutinate	White to pale bluish gray when fresh, bluish gray to ash gray when dry	–	Cylindrical to allantoid	4–5 × 1.5–2	–	This study
*C.nothofagicola*	Australia	Annual, Pileate to effused-reflexed	Buff to olivaceous buff when fresh, pale mouse gray to buff yellow when dry; tomentose	White to cream when fresh, cream to buff yellow when dry	–	Cylindrical to allantoid	3.8–5 × 1–1.7	–	Liu et al. (2021)
*C.piceicola*	China	Annual, Pileate	Cream to clay buff, with bluish gray zones when fresh, light grayish-brown when dry; velutinate	White with a bluish tint when fresh, cream when dry	–	Allantoid	4–4.5 × 0.9–1.3	–	Shen et al. (2019)
*C.populi*	USA	Annual, Pileate to effused-reflexed	White to cream, pale ochraceous to grayish, rarely with bluish flecks or indistinct zones; glabrous	White to cream when fresh, in older and dry specimens with a light bluish-grayish tint	–	Cylindrical to allantoid	4.2–5.6 × 1–1.3	+	Miettinen et al. (2018)
*C.rigidus*	China	Annual, Pileate	Buff yellow to clay buff when fresh, olivaceous buff to grayish brown when dry; glabrous	White to cream when fresh, buff yellow to pinkish buff when dry	–	Cylindrical to allantoid	3.7–4.2 × 0.9–1.3	–	Liu et al. (2022)
*C.simulans*	Finland	Annual, effused-reflexed to resupinate	White to cream when fresh, blue, grayish or pale ochraceous when dry; glabrous	White to cream when fresh, in older and dry specimens with a light bluish-grayish tint	+	Cylindrical to allantoid	4.4–6.3 × 1.3–1.8	+	Miettinen et al. (2018)
*C.subcaesius*	France	Annual, Pileate to effused-reflexed	White to ochraceous with a slight grayish to bluish tint in spots and streaks; glabrous	White to pale gray	–	Allantoid	4–5 × 1–1.2	–	Ryvarden and Melo (2017)
*C.subhirsutus*	China	Annual, Pileate	Pale mouse-gray and cream zones when fresh, cream to buff when dry; hirsute	White when fresh, pinkish buff to honey yellow when dry	–	Allantoid	4–4.5 × 0.9–1.3	+	Shen et al. (2019)
*C.submicroporus*	China	Annual, Pileate	Cream to pinkish buff when fresh, buff to buff yellow when dry; velutinate	White to smoky gray when fresh, buff to olivaceous buff when dry	–	Allantoid	3.6–4.7 × 1–1.3	+	Liu et al. (2021)
*C.subungulatus*	China	Annual, Pileate	Pale mouse-gray to ash-gray when fresh, dark-gray to mouse-gray when dry; glabrous	White to cream when fresh, cream to pinkish buff when dry	–	Cylindrical to allantoid	4.5–5.2 × 1.1–1.4	–	Liu et al. (2022)
*C.subviridis*	Mexico	Annual, Pileate	Pale ochraceous, ochraceous to grayish; glabrous	White to cream, in older and dry specimens with a light bluish-grayish tint	–	Cylindrical to allantoid	3.8–4.5 × 1–1.3	+	Miettinen et al. (2018)
***C.tabuliformis***	China	Annual, pileate	Cream to buff at the base, grayish blue at the margin when fresh, olivaceous buff to ash gray when dry; hirsute	White to sulphur yellow when fresh, cream, pale cinnamon buff to pale mouse-gray when dry	–	Cylindrical to allantoid	4.3–5.5 × 1.5–2	–	This study
*C.tenuicontextus*	China	Annual, Pileate	Cream to pinkish buff with a little blue tint when fresh, light vinaceous gray to pale mouse-gray when dry; velutinate	White to cream when fresh, pinkish buff to buff when dry	–	Allantoid	3.8–4.3 × 0.8–1.2	–	Liu et al. (2022)
*C.tenuis*	China	Annual, Pileate to effused-reflexed	Buff to olivaceous buff when fresh, cream to olivaceous buff when dry; tomentose	White to cream when fresh, buff yellow to pinkish buff when dry	–	Cylindrical, slightly curved	4.7–6 × 1.3–2	+	Liu et al. (2021)
*C.tricolor*	China	Annual, Pileate	Light grayish brown with bluish gray zones when fresh, grayish brown when dry; velutinate	White when fresh, cream to buff when dry	–	Allantoid	4–4.8 × 0.8–1.2	+	Shen et al. (2019)
*C.ungulatus*	China	Annual, Pileate	Olivaceous buff, pinkish buff, cream to ash-gray and white zones when fresh, slightly darkening when dry; glabrous	White when fresh, cream when dry	–	Allantoid	4.5–5 × 0.9–1.2	–	Shen et al. (2019)
*C.yana*e	Russia	Annual, effused-reflexed to resupinate	White to cream, pale ochraceous or bluish to deep brown; glabrous	White, with a light to strong bluish-grayish tint	–	Cylindrical to allantoid	4.3–5.8 × 1.2–1.6	+	Miettinen et al. (2018)

**Bold** = new taxa. Abbreviations used: + = present, – = Absent.

#### ﻿ Cyanosporus linzhiensis

Taxon classificationFungiPolyporalesPolyporaceae

Y.C. Dai, Chao G. Wang, Yuan Yuan & Ghobad-Nejhad sp. nov.

936D5CDD-D979-583D-AA55-A9DC8ED15EEC

853174

Figs 2 , 3

##### Holotype.

China. • Xizang Autonomous Region: Nyingchi, Zayü County, 27 Oct. 2023, on fallen branch of *Pinusyunnanensis*, Dai 27023 (BJFC 044575, GenBank: ITSPP479782, LSU PP479804, mtSSUPP510197, nrSSU PP488289, RPB1 PP526259, RPB2 PP526268).

##### Etymology.

In reference to the species being found in Linzhi (Nyingchi) of Xizang Autonomous Region, southwest China.

##### Diagnosis.

*Cyanosporuslinzhiensis* is characterized by their pileate basidiomata with a bluish tint and azonate pileal surface when fresh and dry, white to pale bluish gray pore surface when fresh, pores angular to irregular, 5–6 per mm, cystidioles fusoid and basidiospores allantoid, 4–5 × 1.2–1.5 µm.

***Basidiomata*** annual, pileate, soft and without odor or taste when fresh, becoming soft corky to fragile upon drying; pileus flabelliform, up to 3 cm, 3.5 cm wide and 8 mm thick at the base. Pileal surface white, somewhat with a bluish tint when fresh, becoming white to pinkish buff when dry, velutinate, azonate. Hymenophore white to pale bluish gray when fresh, becoming pinkish buff to honey yellow and with a blue tint upon drying; sterile margin almost absent; pores angular to irregular, 5–6 per mm, with thin dissepiments becoming lacerate. Context white, soft corky, up to 5 mm thick. Tubes concolorous with pore surface, soft corky to fragile when dry, up to 3 mm long. Context and tubes turn dark olive green in KOH.

**Figure 2.**
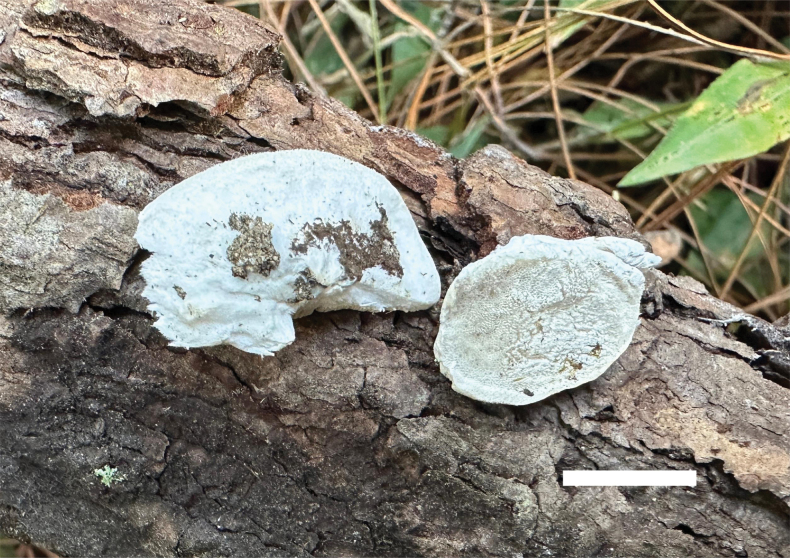
Basidiomata of *Cyanosporuslinzhiensis* (Dai 27023, holotype). Scale bar: 1 cm.

***Hyphal system*** monomitic; hyphae clamped, hyaline, slightly thick-walled, with a wide lumen, smooth; in the context frequently branched, more or less flexuous, loosely interwoven, 3–5 µm in diam; in the tubes unbranched, straight, subparallel along the tubes, agglutinated, 2–3.5 µm in diam.

**Figure 3.**
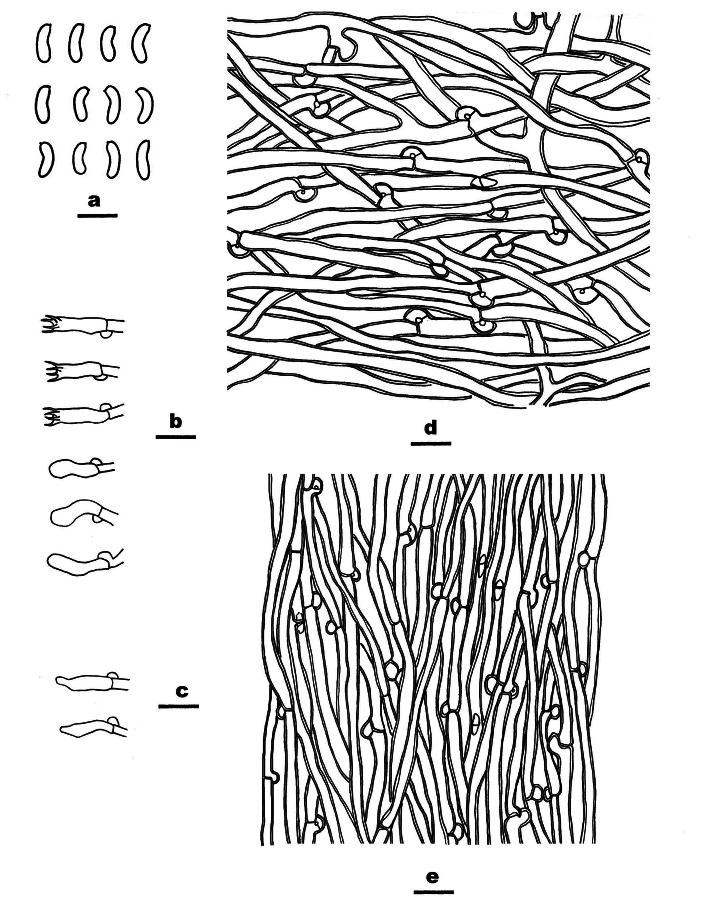
*Cyanosporuslinzhiensis* (Dai 27023, holotype,) **A** basidiospores **B** basidia and basidioles **C** cystidioles **D** hyphae from context **E** hyphae from trama. Scale bars: 5 µm (**A**); 10 µm (**B−E**).

***Cystidia*** absent, but cystidioles fusoid are present, thin-walled, 12–13.5 × 3 µm.

***Basidia*** clavate, 9–13 × 4–5 µm, with basal clamp and four sterigmata.

***Basidiospores*** allantoid, 4–5 × 1.2–1.5 µm, L = 4.5 µm, W = 1.4 µm, Q= 3.1–3.5 (n = 60/2), thin-walled, smooth, hyaline, IKI−, CB−.

##### Type of rot.

Brown rot.

##### Additional specimen examined.

China. • Xizang Autonomous Region: Nyingchi, Bomê County, 27 Oct. 2023, on fallen angiosperm trunk, Dai 27141 (BJFC 044575, GenBank: ITSPP479781, LSU PP479803, mtSSUPP510196, nrSSU PP488288, RPB1 PP526258, RPB2 PP526267).

#### ﻿ Cyanosporus miscanthi

Taxon classificationFungiPolyporalesPolyporaceae

Y.C. Dai, Chao G. Wang, Yuan Yuan & Ghobad-Nejhad sp. nov.

93DC27C5-16C8-5653-865C-946184177AD7

853175

Figs 4 , 5

##### Holotype.

China. • Xizang Autonomous Region: Nyingchi, Medog County, 24 Oct. 2023, on dead *Miscanthus*, Dai 26687 (BJFC 044237, GenBank: ITSPP479786, LSU PP479808, mtSSUPP510201, nrSSU PP488293, RPB1 PP526263, RPB2 PP526272, TEF1 PP526277).

##### Etymology.

In reference to *Miscanthus* the genus where this species was found.

##### Diagnosis.

*Cyanosporusmiscanthi* is characterized by effused-reflexed to pileate tiny basidiomata, slightly concentrically zonate pileal surface, white to pale bluish gray pore surface when fresh, angular pores, 7–9 per mm, fusoid cystidioles and cylindrical to allantoid basidiospores, 4–5 × 1.5–2 µm.

**Figure 4.**
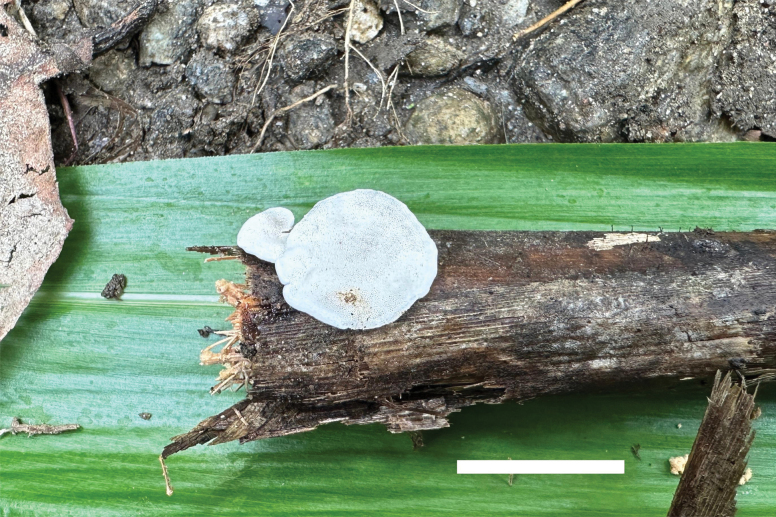
Basidiomata of *Cyanosporusmiscanthi* (Dai 26687, holotype). Scale bar: 1 cm.

***Basidiomata*** annual, effused-reflexed to pileate, soft and without odor or taste when fresh, becoming fragile to soft corky upon drying, up to 1 cm long and 0.8 cm wide when resupinate; pileus semicircular, projecting up to 0.8 cm, 1.2 cm wide and 1.2 mm thick at the base. Pileal surface white, pale bluish gray to bluish green when fresh and dry, velutinate, slightly concentrically zonate when dry; margin sharp, slightly curved when dry. Hymenophore poroid, white to pale bluish gray when fresh, becoming bluish gray to ash gray when dry; sterile margin almost absent; pores angular, 7–9 per mm; dissepiments thin, entire to slightly lacerate. Context white, soft corky, up to 0.3 mm thick. Tubes concolorous with pore surface, fragile to soft corky when dry, up to 0.9 mm long. Context and tubes turn dark olive green in KOH.

**Figure 5.**
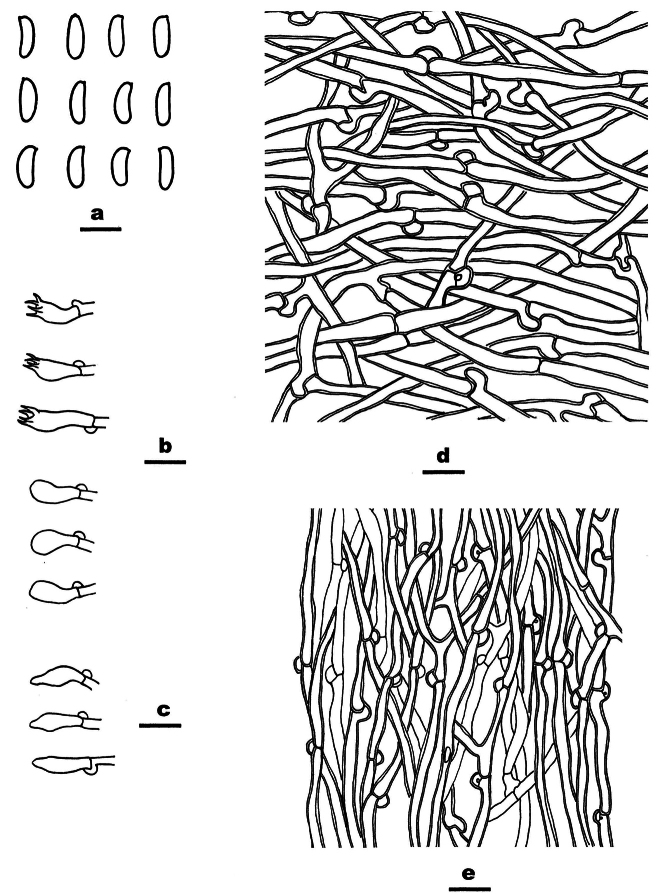
*Cyanosporusmiscanthi* (Dai 26687, holotype) **A** basidiospores **B** basidia and basidioles **C** cystidioles **D** hyphae from context **E** hyphae from trama. Scale bars: 5 μm (**A**); 10 μm (**B−E**).

***Hyphal system*** monomitic; hyphae clamped, hyaline, slightly thick-walled, smooth, with a wide lumen, frequently branched, more or less flexuous, in the context loosely interwoven, 3–4.5 µm in diam in the tubes subparallel along the tubes, agglutinated, 2.5–3 µm in diam.

***Cystidia*** absent, but cystidioles fusoid present, thin-walled, 11–15 × 4 µm.

***Basidia*** clavate, 9–13 × 4–5 µm, with four sterigmata and a basal clamp connection.

***Basidiospores*** cylindrical to allantoid, 4–5(–5.5) × 1.5–2 µm, L = 4.2 µm, W = 1.9 µm, Q = 2.2–2.4 (n = 120/4), hyaline, thin-walled, IKI−, CB−.

##### Type of rot.

Brown rot.

##### Additional specimens examined.

China. • Xizang Autonomous Region: Nyingchi, Medog County, 24 Oct. 2023, on dead *Miscanthus*, Dai 26684 (BJFC044234, ITSPP479784, LSU PP479806, mtSSUPP510199, nrSSU PP488291, RPB1 PP526261, RPB2 PP526270, TEF1 PP526276); Dai 26695 (BJFC044245, ITSPP479787, LSU PP479809, mtSSUPP510202, nrSSU PP488294, RPB1 PP526264, RPB2 PP526273, TEF1 PP526278); Dai 26689 (BJFC044239, ITSPP479783, LSU PP479805, mtSSUPP510198, nrSSU PP488290, RPB1 PP526260, RPB2 PP526269, TEF1 PP526275); Dai 26701 (BJFC044251, ITSPP479785, LSU PP479807, mtSSUPP510200, nrSSU PP488292, RPB1 PP526262, RPB2 PP526271).

#### ﻿ Cyanosporus tabuliformis

Taxon classificationFungiPolyporalesPolyporaceae

Y.C. Dai, Chao G. Wang, Yuan Yuan & Ghobad-Nejhad sp. nov.

8022981E-7536-5C66-A1D6-85E1C7E5E358

853176

Figs 6 , 7

##### Holotype.

China. • Shanxi Province: Changzhi, Qinyuan County, Taiyueshan Forest Park, 31 Aug. 2023, on fallen branch of *Pinustabuliformis*, Dai 26063 (BJFC 043612, Genbank: ITSPP479788, LSU PP479810, mtSSUPP510203, nrSSU PP488295, RPB1 PP526265, RPB2 PP526274, TEF1 PP526279).

##### Etymology.

In reference to the specific epithet of the substrate, *Pinustabuliformis* in which this species was found.

##### Diagnosis.

*Cyanosporustabuliformis* is characterized by a pileate basidiomata with cream, buff to grayish blue and hirsute azonate pileal surface when fresh, angular pores, 4–5 per mm, fusoid cystidioles, and cylindrical to allantoid basidiospores, 4.3–5.5 × 1. 5–2 μm.

**Figure 6.**
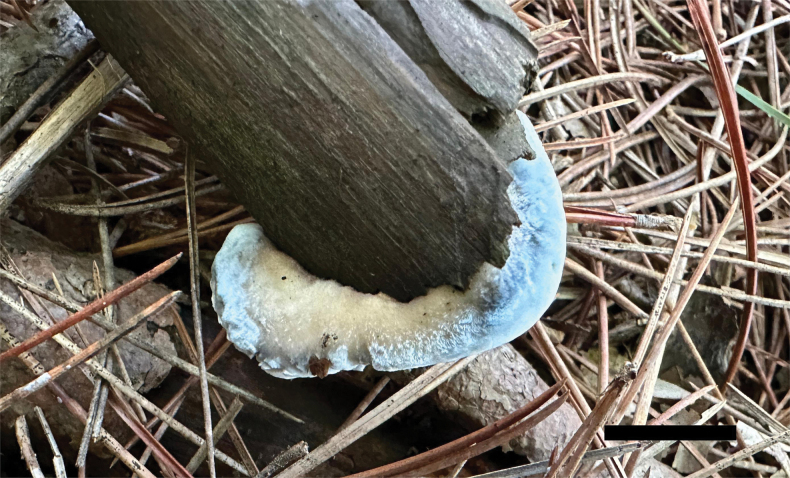
Basidiomata of *Cyanosporustabuliformis* (Dai 26063, holotype). Scale bar: 1 cm.

***Basidiomata*** annual, pileate, soft and without odor or taste when fresh, becoming more or less fragile to corky upon drying. Pileus flabelliform, projecting up to 1.5 cm, 3.5 cm wide and 5 mm thick at the base. Pileal surface cream to buff at the base, grayish blue at the margin when fresh, becoming olivaceous buff to ash gray upon drying, hirsute, azonate when dry; margin blunt. Hymenophore poroid, white to sulphur yellow when fresh, unchanged when bruised, becoming cream, pale cinnamon buff to pale mouse gray upon drying; sterile margin white when fresh, cream to buff when dry, up to 0.2 mm wide; pores angular to irregular, 4–5 per mm, with thin dissepiments, becoming lacerate. Context white, soft corky, up to 2 mm thick. Tubes concolorous with pore surface, fragile to soft corky when dry, up to 3 mm long.

**Figure 7.**
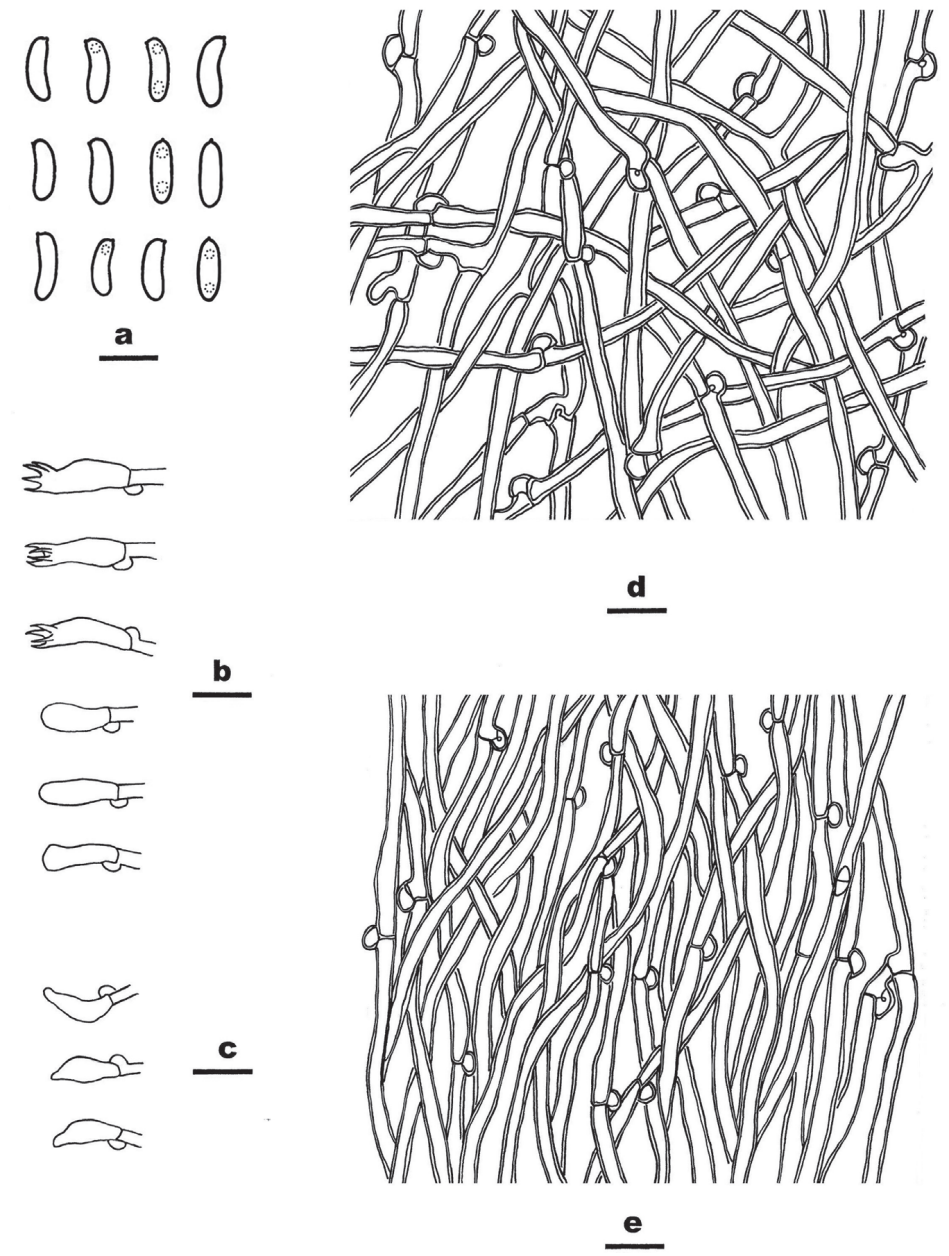
*Cyanosporustabuliformis* (Dai 26063, holotype) **A** basidiospores **B** basidia and basidioles **C** cystidioles **D** hyphae from context **E** hyphae from trama. Scale bars: 5 µm (**A**); 10 µm (**B−E**).

***Hyphal system*** monomitic, hyphae clamped, hyaline, slightly thick-walled with a wide lumen, in the context frequently branched, straight, distinctly interwoven, 3–4 µm in diam; in the tubes rarely branched, more or less flexuous, subparallel along the tubes, agglutinated, 2.8–3.5 µm in diam.

***Cystidia*** absent, but cystidioles fusoid present, 10–12 × 4 µm.

***Basidia*** clavate, 13–16 × 4.5–5 µm, with basal clamp and four sterigmata.

***Basidiospores*** cylindrical to allantoid, 4.3–5.5 × 1. 5–2 μm, L = 4.8 µm, W = 1.9 µm, Q = 2.6 (n = 60/2), hyaline, thin-walled, sometimes with one or two small guttules, IKI−, CB−.

##### Type of rot.

Brown rot.

##### Additional specimen examined.

China. • Shanxi: Changzhi, Qinyuan County, Taiyueshan Forest Park, 31 Aug. 2023, on fallen branch of *Pinustabuliformis*, Dai 26066 (BJFC 043615, Genbank: ITSPP479789, LSU PP479811, mtSSUPP510204, nrSSU PP488296, RPB1 PP526266, TEF1 PP526280).

## ﻿Discussion

The *Cyanosporus* was established by McGinty in1909 with one species *C.caesius*. Recently, Papp (2014) based on morphological characters, proposed the subgenus Postiasubg.Cyanosporus for the *P.caesius* complex. Miettinen et al. (2018) studied the *Postiacaesia* complex using sequences from two DNA loci, ITS and TEF1 selected a neotype of *P.caesia* (LY BR-6776 collected from Germany) from type locality and described ten new species in *Postia*. However, *Cyanosporus* as an independent genus was raised again in recent studies (Shen et al. 2019; Liu et al. 2021, 2022, 2023). In our study, samples of 38 *Cyanosporus* species including three new species formed a strongly supported clade distinguished from *Postia* (Fig. 1).

*Cyanosporus* is a cosmopolitan genus causing a brown rot in different angiosperm and gymnosperm wood. Out of 38 species, currently 26 species are recorded in China. *Cyanosporus* usually has effused-reflexed to pileate poroid basidiomata with a bluish tint and thin- to slightly thick-walled basidiospores distinguished from other genera of Postiaceae (Liu et al. 2023).

*Cyanosporuslinzhiensis* is phylogenetically related to *C.magnus* (Miettinen) B.K. Cui & Shun Liu, and both species have pileate basidiomata with white, velutinate and azonate pileal surface, almost the same size of pores (4–5 per mm in *C.magnus* vs. 5–6 per mm in *C.linzhiensis*, Miettinen et al. 2018), and they are recorded in China. However, the latter has distinct white pileal margin and narrower basidiospores (3.6–4.4 × 1–1.2 µm vs. 4–5 × 1.2–1.5 µm, Miettinen et al. 2018). *Cyanosporuscaesiosimulans* and *C.livens* are similar to *C.linzhiensis* by white velutinate pileal surface, almost the same pores (5–7 per mm in *C.caesiosimulans*; 4–6 per mm in *C.livens*; 5–6 per mm in *C.linzhiensis*, Miettinen et al. 2018) and allantoid basidiospores of about the same size (4.2–5.5 × 1.1–1.4 µm in *C.caesiosimulans*; 4.1–5.7 × 1.1–1.5 µm in *C.livens*; 4–5 × 1.2–1.5 µm in *C.linzhiensis*, Miettinen et al. 2018). However, the former two are not currently distributed in China, and unrelated to *C.linzhiensis* in phylogeny.

*Cyanosporusmiscanthi* and *C.rigidus* B.K. Cui & Shun Liu are phylogenetically related (Fig. 1), but they are different in morphology. The latter has rigid basidiomata when dry, buff yellow to clay buff and distinct concentrically zonate pileal surface when fresh, buff-yellow to pinkish buff pore surface when dry, the absence of cystidioles and smaller basidiospores (3.7–4.2 × 0.9–1.3 µm vs. 4–5 × 1.5–2 µm, Liu et al. 2022). *Cyanosporusnothofagicola* B.K. Cui, Shun Liu & Y.C. Dai, *C.tenuis* B.K. Cui, Shun Liu & Y.C. Dai and *C.miscanthi* share effused-reflexed to pileate basidiomata, soft corky to fragile when dry, angular pores, white, pale mouse gray to pale bluish gray pore surface when fresh, and fusoid cystidioles. However, *C.nothofagicola* has narrower basidiospores (3.8–5 × 1–1.7 µm vs. 4–5 × 1.5–2 µm, Liu et al. 2021), and is grown on *Nothofagus* occurring in Australia. *Cyanosporustenuis* also has tiny basidiomata, but it has wider contextual hyphae (2.6–7 µm in diam. vs. 3–4.5 µm in diam.), larger basidia (18–28 × 3.7–6 µm vs. 9–13 × 4–5 µm) and relatively larger basidiospores (4.7–6 × 1.3–2 µm vs. 4–5 × 1.5–2 µm, Liu et al. 2021). In addition, they form independent lineages in the phylogeny (Fig. 1).

*Cyanosporustabuliformis* and *C.auricomus* (Spirin & Niemelä) B.K. Cui & Shun Liu form a sister group without strong support. They share the pileate basidiomata with hirsute and azonate pileal surface, almost the same size of pores (4–6 per mm vs. 4–5 per mm, Miettinen et al. 2018) and basidiospores (4.4–5.6 × 1.5–1.8 µm vs. 4.3–5.5 × 1.5–2 µm, Miettinen et al. 2018), and growth on gymnosperm wood. However, the latter has bright yellow pore surface when fresh, green when bruised (Miettinen et al. 2018); moreover, their sister relationship lacks strong support hinting at them being two distinct species. *Cyanosporuscyanescens* (Miettinen) B.K. Cui & Shun Liu has pileal surface with cream to pale ochraceous color at the base, grayish blue at the margin when fresh, and it is somewhat similar to *C.tabuliformis*, yet its slimmer basidiospores (4.7–6.1 × 1.1–1.6 µm vs. 4.3–5.5 × 1.5–2 µm, Miettinen et al. 2018) make it different from *C.tabuliformis*. In addition, *C.cyanescens* is distantly related to *C.tabuliformis* in our phylogeny (Fig. 1).

Although extensive studies on Chinese polypores have been carried out recently, and more than 1000 species were reported (Dai 2009; Cui et al. 2019; Dai et al. 2021; Wu et al. 2022a, 2022b; Mao et al. 2023; Wang et al. 2023; Yuan et al. 2023; Zhang et al. 2023; Zhou et al. 2023; Zhao et al. 2024), the richness of this group fungi is still not well recognized, especially in the southwest China. In this paper two species of *Cyanosporus* are described from Xizang (Tibet, southwest China) demonstrate that more taxa will be described after further investigations in the virgin forests of Xizang.

## Supplementary Material

XML Treatment for Cyanosporus linzhiensis

XML Treatment for Cyanosporus miscanthi

XML Treatment for Cyanosporus tabuliformis

## ﻿Additional information

### Author ORCIDs

Chao-Ge Wang https://orcid.org/0000-0003-4381-5720

Shun Liu https://orcid.org/0000-0001-9261-4365

Masoomeh Ghobad-Nejhad https://orcid.org/0000-0002-7807-4187

Hong-Gao Liu https://orcid.org/0000-0002-9508-3245

Yu-Cheng Dai https://orcid.org/0000-0002-6523-0320

Yuan Yuan https://orcid.org/0000-0001-6674-9848

### Data availability

All of the data that support the findings of this study are available in the main text.
